# Hazardous Petroleum Sludge-Derived Nitrogen and Oxygen Co-Doped Carbon Material with Hierarchical Porous Structure for High-Performance All-Solid-State Supercapacitors

**DOI:** 10.3390/ma14102477

**Published:** 2021-05-11

**Authors:** Xiaoyu Li, Mingyang Zhang, Zhuowei Tan, Zhiqiang Gong, Peikun Liu, Zhenbo Wang

**Affiliations:** 1 College of Mechanical and Electronic Engineering, Shandong University of Science and Technology, Qingdao 266590, China; lpk@sdust.edu.cn; 2 College of New Energy, China University of Petroleum (East China), Qingdao 266580, China; b16030090@s.upc.edu.cn (Z.T.); wangzhb@upc.edu.cn (Z.W.); 3 School of Thermal Engineering, Shandong Jianzhu University, Jinan 250101, China; 4 State Grid Shandong Electric Power Research Institute, Jinan 250003, China; gongzhiqiang@upc.edu.cn

**Keywords:** petroleum sludge, nitrogen/oxygen co-doping, porous carbon, supercapacitors

## Abstract

Rational design and sustainable preparation of high-performance carbonaceous electrode materials are important to the practical application of supercapacitors. In this work, a cost-effective synthesis strategy for nitrogen and oxygen co-doped porous carbon (NOC) from petroleum sludge waste was developed. The hierarchical porous structure and ultra-high surface area (2514.7 m^2^ g^−1^) of NOC electrode materials could provide an efficient transport path and capacitance active site for electrolyte ions. The uniform co-doping of N and O heteroatoms brought enhanced wettability, electrical conductivity and probably additional pseudo-capacitance. The as-obtained NOC electrodes exhibited a high specific capacitance (441.2 F g^−1^ at 0.5 A g^−1^), outstanding rate capability, and cycling performance with inconspicuous capacitance loss after 10,000 cycles. Further, the assembled all-solid-state MnO_2_/NOC asymmetrical supercapacitor device (ASC) could deliver an excellent capacitance of 119.3 F g^−1^ at 0.2 A g^−1^ under a wide potential operation window of 0–1.8 V with flexible mechanical stability. This ASC device yielded a superior energy density of 53.7 W h kg^−1^ at a power density of 180 W kg^−1^ and a reasonable cycling life. Overall, this sustainable, low-cost and waste-derived porous carbon electrode material might be widely used in the field of energy storage, now and into the foreseeable future.

## 1. Introduction

Utilization of clean and renewable energy is playing an increasingly significant role in green and sustainable development [1,2,3]. Nevertheless, the intermittence or fluctuation features of some clean energy resources, such as wind, solar or wave energy, hinder their full utilization. It is imperative to develop high-performance energy storage (ES) systems. Supercapacitors, which build a bridge between traditional electrostatic capacitors and batteries, have recently been treated as a promising ES device due to their balanced energy storage capacity and power delivery capability as well as their superior long-term durability [4].

Commonly, supercapacitors can be classified into two different types, electric double-layer capacitors (EDLC) and pseudo-capacitors, based on their ES mechanism. Among them, EDLC used carbonaceous electrodes materials to generate capacitance by accumulating charges at the interfaces between an electrode and electrolyte. Meanwhile, the electrode materials of a pseudo-capacitor are transition metal oxides or conduct polymers, which could produce pseudo-capacitance from fast Faradaic reactions [5,6]. Porous carbons with a high surface area are most widely used in commercial EDLCs, because of their favorable chemical stability, electrical conductivity and because they are environmentally friendly [7]. Up to now, many advanced carbon materials, such as activated carbon [8], carbon nanotubes [9,10], carbon nanofibers [11], and graphene and its derivatives [12,13,14,15] have been explored as supercapacitor electrode materials. Of special interest from a sustainable and environmental perspective are carbon materials derived from abundant renewable waste, such as biomass [16,17], plastic [18], gel [19], paper products [20], organic sludge [21], and so on. However, the relative low capacitance performance of waste-based carbon has made it unable to meet the demands of practical applications [22].

To improve the ES properties of carbon electrodes, reasonable structure and composition designs have both been proved to be extremely effective. Firstly, nanostructure design is an efficient way to improve the electrochemical performance of carbon electrodes. The introduced pores of different sizes can play a synergistic role in the ES process. Micro-pores can provide sufficient accessible ion sites, which results in a considerable capacitance effect. Meso- and macro-pores play the roles of fast transportation routes and ion-buffering reservoirs for electrolyte ions, which result in a high rate capability [23]. Apart from the interconnected pore structures, some special heteroatoms such as N [24,25], O [26], B [27], S [28], F [29], and P [30] doped into the porous carbon matrix would also be beneficial for capacitance behavior. Among these, N-doping and O-doping are considered promising approaches due to their wide range of sources. The presence of O in the carbon matrix has been proved to enhance the surface wettability of carbon electrodes to aqueous electrolyte, which accelerates the effective charge accumulation on the interfaces and introduces additional active sites for redox reactions. Additionally, N-doping has also been confirmed to improve electrical conductivity and provide additional Faradaic capacitance effects. Therefore, it is worth to explore the improved capacitance performance of N/O co-doped carbon electrodes [31].

To realize improvements to the carbon porous structure, some effective approaches have been obtained, such as template methods, self-template methods, and various activation methods. Generally, the current strategy to achieve the effective doping of N or O into a carbon skeleton could be divided into two categories, direct synthesis and post-synthesis, respectively [32]. The former attempts to use the N-enriched, O-enriched, or N/O-co-enriched carbon precursor for pyrolysis and activation treatment. The latter method involves the penetration of N/O atoms into the already constructed carbon skeleton, always in the forms of a surface modification [31]. In fact, most methods for both porous structure optimization and heteroatom doping regulation have their own defects, such as a severe demand for raw materials, laborious and costly synthesis procedures, implacable requirements on the environment, and so on.

As mentioned above, exploring waste-based heteroatom-doping carbon materials are promising and challenging. Petroleum sludge (PS), a common solid waste produced in the petroleum industry, undermines environmental protection efforts until a proper disposal of crude oil components is achieved [33]. PS yield was gradually expanding with the development of petroleum industry. From another perspective, the fuels contained in PS are also an attractive renewable and resourceful energy. There have been some studies about PS-based carbonaceous materials for present applications in wastewater treatment [34], CO_2_ capture [35], and electrode materials [36]. However, complex synthesis procedures and relatively poor structure performance obviously hinder their large-scale production and application. Therefore, there is an urgent demand to explore a high-efficiency strategy to convert PS into higher-performance carbon materials using simple, green and economical methods.

Herein, we report a novel N/O-co-doped carbon material with a high electrochemical capacitance performance from PS produced with a two-step process that simultaneously realizes porosity regulation and the self-doping of heteroatoms. The PS was firstly pre-carbonized and acid-trimmed to form a basic carbon skeleton, and then co-pyrolyzed with KOH and urea to add O and N atoms in the structure. The hierarchical porous structure, high surface area, and numerous N/O heteroatoms of the unique NOC obtained under optimal conditions allowed it to deliver a surprising specific capacitance and outstanding long-term cycling ability. Moreover, the assembled ASC devices using NOC as an anode and MnO_2_ nanoparticles as the cathode also showed excellent energy density and long cycling stability.

## 2. Materials and Methods

### 2.1. Materials Preparation

**Scheme 1:**
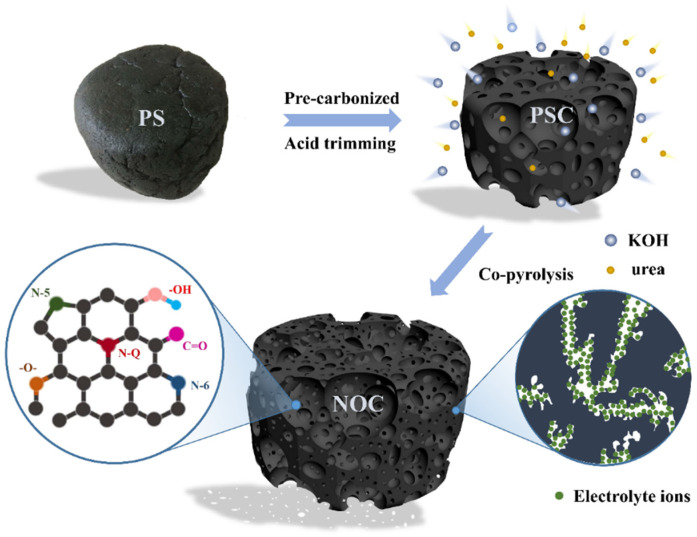
Synthesis procedure of NOC materials.

The PS sample used in this work is the sediment of a petroleum storage tank from Dongying Oil Depot (Dongying, Shandong, China). The proximate and ultimate analyses of PS are listed in Table S1. The brief synthesis procedure of the NOC material is shown in Scheme 1. The PS sample was dried overnight at 80 °C and pre-carbonized for 2 h at 700 °C under a N_2_ (99.9%) atmosphere in a tube furnace. The received pyrolysis char was boiled in an excess of 1 M HF solution in a fume hood to avoid inorganic mineral impurities. After that, solid products, namely petroleum sludge carbon (PSC), were rinsed with water and then dried and collected. Then, a mixture of 0.2 g PSC, 0.6 g KOH and 1.0 g urea was dissolved and dispersed in deionized water by sonication. After drying at 80 °C for 10 h in vacuum, the obtained solid product was ground and annealed in a fix-bed tube reactor under Ar flow for 2 h at 600, 700, and 800 °C, respectively. After being washed by 1 M HCl and sufficient deionized water, NOC products were obtained. According to the different preparation temperature, the obtained samples were named NOC-600, NOC-700 and NOC-800, respectively. For comparison, the porous carbon was annealed at 700 °C using the same steps while that without urea was designated as PAC-700.

### 2.2. Electrochemical Test

As-prepared NOC materials were mixed with polyvinylidene-fluoride and carbon black at a mass ratio of 8:1:1 into N-methyl-2-pyrrolidone to form a slurry, which was then coated onto nickel foam with an area of 1 cm^2^ to fabricate a working electrode. Then, a traditional three-electrode system was assembled with Hg/HgO as the reference electrode, Pt foil as the counter electrode and 6 M KOH solution as the electrolyte, respectively. The cyclic-voltammetry (CV) test and galvanostatic charging–discharging (GCD) test were carried out at −1.0–0 V (vs. Hg/HgO) on a CHI-660E electrochemical workstation (Shanghai Chenhua Science Technology Corp., Ltd., Shanghai, China), and electrochemical impedance spectroscopy (EIS) measurements were performed at a frequency range of 100 kHz to 0.01 Hz on a Reference 600 electrochemical workstation (Gamry, Warminster, PA, USA).

To assemble the two-electrode supercapacitor device, MnO_2_ nanoparticles were uniformly grown on Ni foam as a cathode using the electrodeposition method described in detail in the Supplementary Materials. Then, the NOC anode and MnO_2_ cathode were separated by PVA/LiCl gel electrolyte and pressed together to fabricate the all-solid-state ASC device.

The calculation method for the specific capacitance, energy densities and power densities are also provided in the Supplementary Materials.

## 3. Results

### 3.1. Structural Characterization

**Figure 1:**
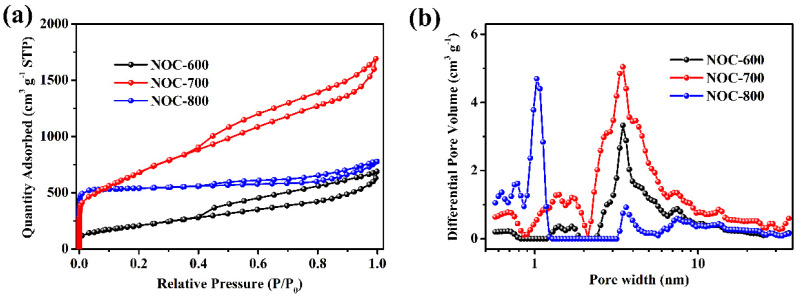
N_2_ adsorption–desorption isotherms (**a**) and the pore size distributions (**b**) of NOCs materials.

In general, the capacitance performances of electrode materials are highly related to their pore structures and specific surface areas. N_2_ adsorption–desorption tests of NOC samples were carried out and are illustrated in Figure 1a. NOC-600 and NOC-700 both exhibited combined I/IV type isotherms with obvious H4 hysteresis loops [37], which represents the presence of micro- and meso-pores in the materials. Meanwhile, the isotherm of NOC-800 conformed to the properties of typical type I isotherms, indicating the dominant position of micro-pores. BET surface area could be calculated using these isotherms data, as listed in Table S2. NOC-700 possesses the highest specific surface area of 2514.7 m^2^ g^−1^, of which the micro-pore contribution rate is only 42%. However, the surface area of NOC-600 and NOC-800 are 767.7 and 1847.0 m^2^ g^−1^, respectively, indicating that the temperature has a significant influence on pore formation and evolution. As shown in Figure 1b, the pores size distributions of these NOCs were calculated by applying the NLDFT method. Due to the fact that the activation action of KOH cannot be fully carried out [38] at a lower temperature of 600 °C, the main contribution of the surface area comes from the meso-pores in NOC-600. As the temperature increased to 700 °C, the pore expansion effect of KOH gradually increased, and the NOC-700 exhibited a hierarchical porous structure. The volume of both micro-pores and meso-pores were obviously increased. However, when the temperature reached 800 °C, the carbon structure of the original PSC precursor was destroyed, and then a very large amount of micro-pores was introduced and gradually dominated the carbon skeleton [39].

**Figure 2:**
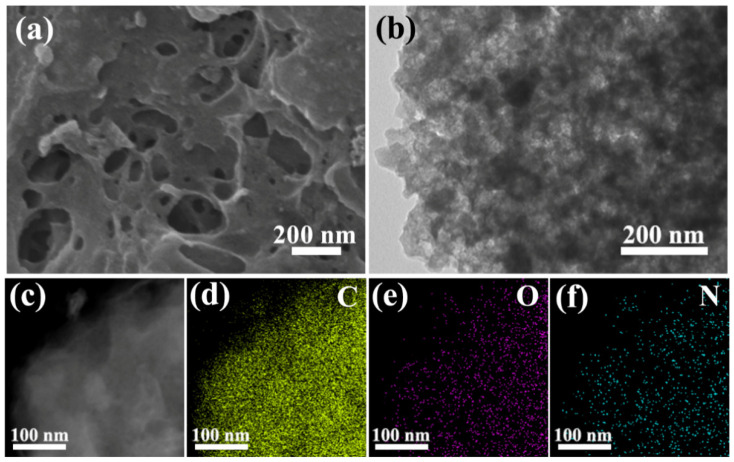
SEM and TEM images (**a**–**c**) of NOC-700 and the elemental mapping images of C (**d**), O (**e**), and N (**f**).

In order to analyze the morphological evolution of NOCs, SEM images of the obtained samples were observed. As shown in Figure 2a, NOC-700 exhibits a porous structure that may be caused by acid pickling and KOH activation [36]. In particular, the obvious macro-pores would act as buffering reservoirs for electrolyte ions, which favors a higher capacitance rating performance. As a comparison, although NOC-600 (Figure S1a), NOC-650 (Figure S2a), NOC-750 (Figure S2b), and NOC-800 (Figure S1b) also reveal a certain degree of porosity, there are still significant differences, as observed. Consistent with the previous analyses, the pore structures of NOC-600 and NOC-650 are not as obvious as that of NOC-700, due to the lack of a KOH activation effect. At the same time, with an increase in temperature, the surface of NOC-750 and NOC-800 become smoother, because the higher activation temperature brings the excess reaction of KOH and the thin carbon walls on the surface of PSC (Figure S1c), thereby causing the surface carbon to shrink into a spherical shape resulting in a poor porous characteristic. In addition, the morphologies of PAC-700 (Figure S1d) and NOC-700 have no obvious differences, and plentiful nanopores are interconnected with each other. This means that the doping of N has little effect on the porous structure.

To further determine the micro-structure of NOC-700, TEM observations were carried out. It is clear from Figure 2b that white spots on the gray background underline the abundant pores and cracks inside, which could produce a relatively high specific surface area. Figure 2c–f gives the elemental mapping images of NOC-700, indicating that it contains uniform C, O, and N elements.

**Figure 3:**
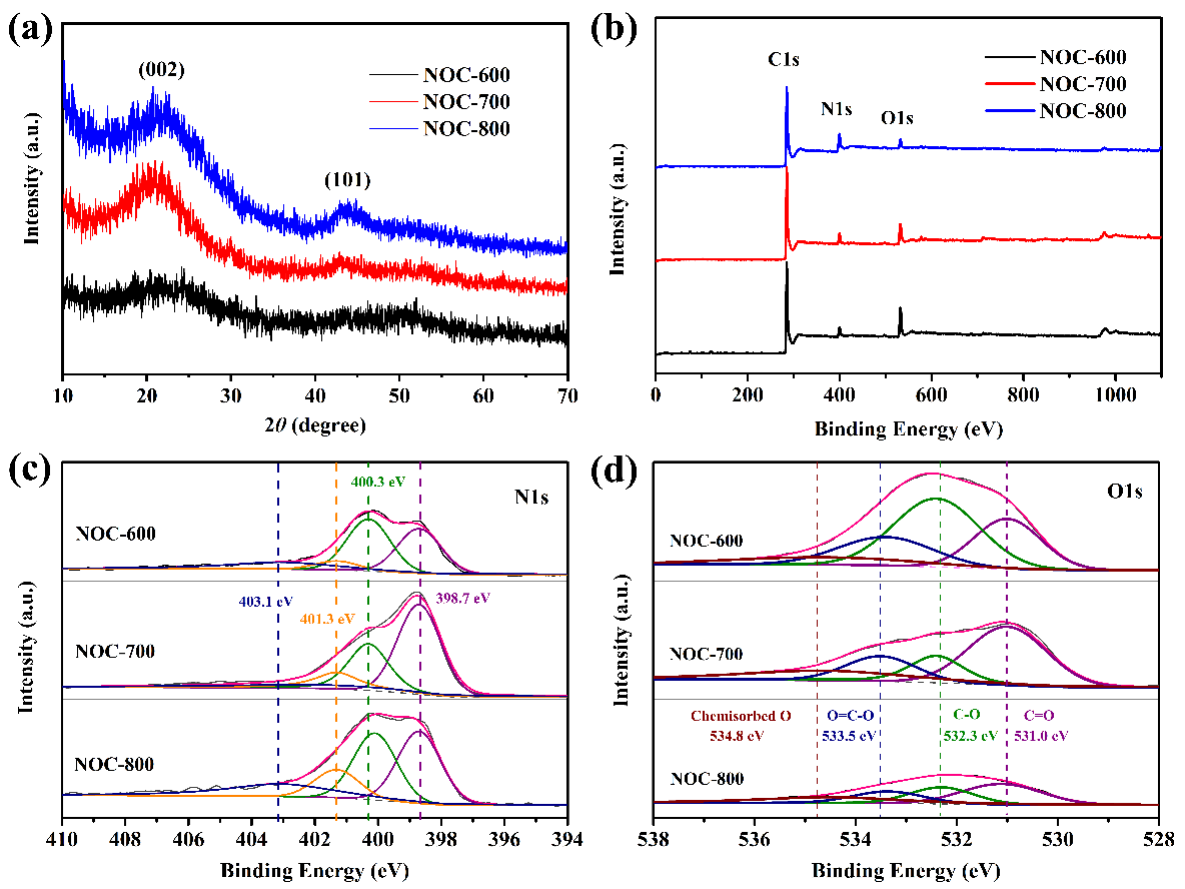
XRD patterns (**a**) and XPS spectra (**b**) of NOCs samples, and the high resolution of N1s (**c**) and O1s spectra (**d**) of NOCs.

XRD patterns of three NOC materials are shown in Figure 3a. Two broad diffraction peaks were found for all the samples at around 24° and 44°, which are attributed to the (002) and (101) planes of turbostratic carbon, respectively. This demonstrates the presence of graphitic carbon in NOC materials. In addition, the (101) peaks are more conspicuous in the patterns as the temperature increases, indicating an increase in the degree of graphitization.

XPS analyses were performed to determine the surface chemical states of NOC materials. Clearly, C1s (285 eV), O1s (532eV), and N1s (400 eV) peaks can be observed in the XPS surveys (Figure 3b), simultaneously confirming the successful N/O co-doping, which is consistent with elemental mapping analyses of the TEM tests. The percentages of C, N, and O in all the NOCs are shown in Table S3. It is obvious that, with the increase in pyrolysis temperature, the N content on the surfaces increased while the O content gradually decreased. In addition, the bulk composition of the NOC samples was tested by an elemental analyzer. As shown in Table S4, there is a difference between the surface and bulk compositions, in which the surface O and N contents are somewhat higher than those detected by elemental analyses, due to the surface treatment with urea during processing.

The high-resolution XPS spectra of C1s, N1s, and O1s for all the samples were analyzed. As shown in Figure S3, the C1s spectra can be deconvoluted into four peaks, located at 284.8 eV, 285.6 eV, 288.0 eV, and 290.1 eV, representing sp^2^-C, sp^3^-C, C-O/C-N and C=O, respectively [40]. In addition, The N1s peaks could be deconvoluted by incorporation of four contribution including pyridinic-N (N-6, 398.7 eV), pyrrolic/pyridone N (N-5, 400.3 eV), quaternary N (N-Q, 401.3 eV), and oxidized pyridine N (N-O, 403.1 eV) as demonstrated in Figure 3c [41,42]. It is believed that N-Q plays a crucial role in the enhancement of the charge transfer in electrode materials. N-6 and N-5 have also been reported to supply pseudocapacitance effects, as listed below [43,44]:

>C=NH_2_ + 2 OH^−^ ↔ >C=NH + 2 *e*^−^ + H_2_O

>C-NH_2_ + 2 OH^−^ ↔ >C-NHOH + 2 *e*^−^ + H_2_O

In addition, the O1s spectra could also be divided into four peaks (Figure 3d) around 531.0 eV, 532.3 eV, 533.5 eV, and 534.8 eV, attributed to C=O, C-O, O=C-O, and chemisorbed O, respectively [45,46]. These O-containing groups have been shown to improve surface wettability and participate in Faradaic reactions in an alkaline electrolyte environment, as follows [41,43]:

>CH-O^−^ + OH^−^ ↔ >C=O + e^−^ + H_2_O

-COOH + OH^−^ ↔ −COOH^−^ + e^−^ + H_2_O

>C-OH + OH^−^ ↔ >C=O + e^−^ + H_2_O

The existence of N/O-containing groups, as listed above, could make NOCs possessing a high affinity to aqueous electrolytes. Therefore, the wettability of NOC electrodes can be enhanced in aqueous electrolytes, easily accelerating ion transport and diffusion into the micro-pores. Both EDLC and pseudo-capacitance can be improved due to the enhanced electrostatic interactions, as well as the increased accessibility of the electrolytes to electroactive species. Furthermore, N/O co-doping especially N-Q species could dramatically enhance the electrical conductivity of NOC samples, which was determined to be 14.8, 8.7 and 9.3 S cm^−1^ for NOC-600, 700, and 800, respectively.

Considering their distinctive features, such as their excellent porous frameworks, high surface areas, and high-level heteroatom doping nature, it is reasonable that such NOC materials could have a great application potential in supercapacitor electrodes.

### 3.2. Capacitance Performance of the NOCs

**Figure 4:**
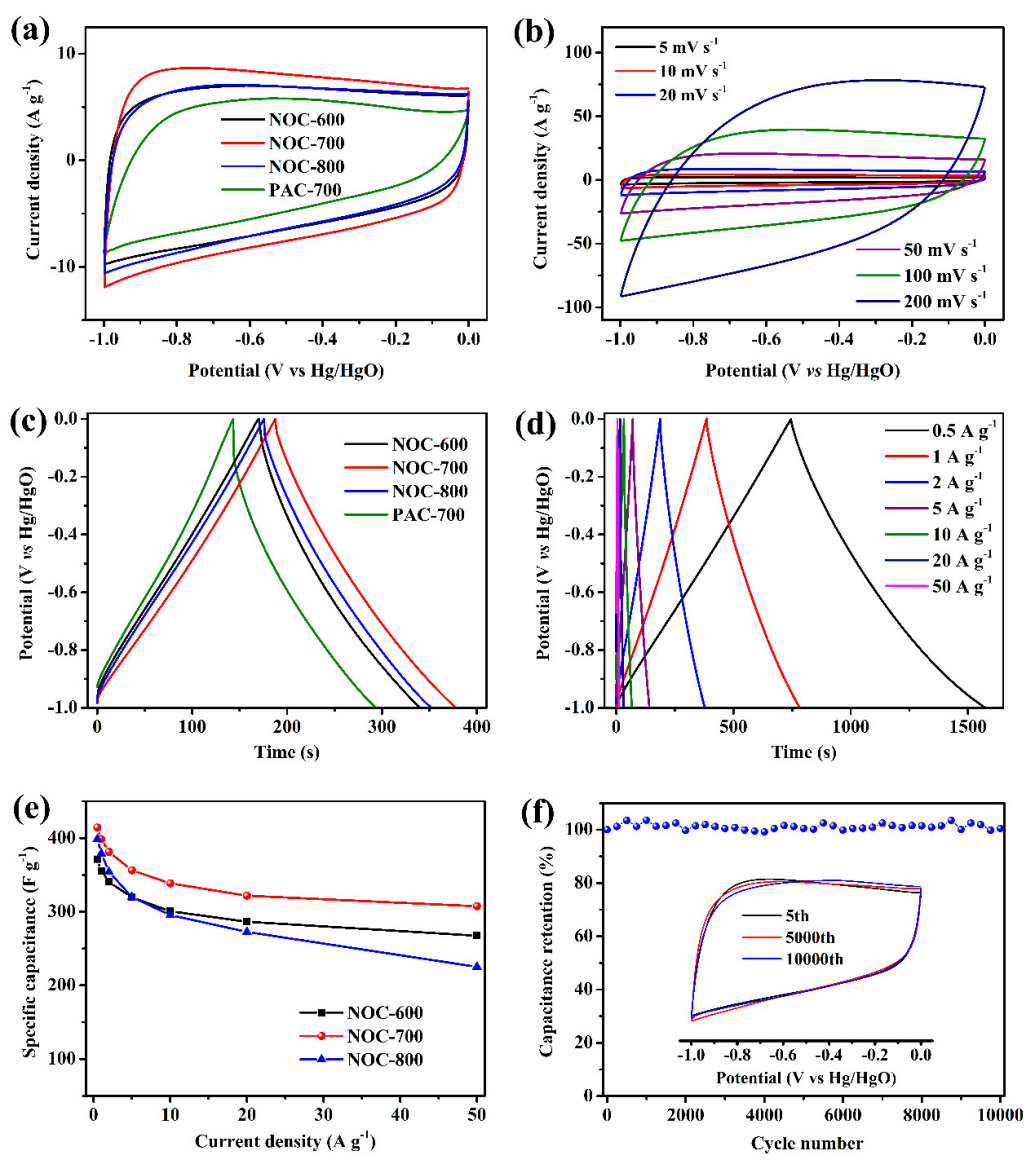
The comparison of CV curves of different electrodes at 20 mV s^−1^ (**a**) and CV curves of NOC-700 at different sweep rates (**b**). The GCD curves of different electrodes at 2 A g^−1^ (**c**), and GCD curves of NOC-700 at different current densities (**d**). The specific capacitance and rate performance (**e**) of different NOC electrodes calculated by GCD curves, and the cycling ability (**f**) of NOC-700 electrode based on CV test. The inset in (**f**) is the 5th, 5000th, and 10,000th cycle of CV tests at 50 mV s^−1^.

The capacitance properties of NOC electrodes were first measured in a traditional half-cell system. For a better comparison, the CV curves of NOCs and PAC electrodes at a sweep rate of 20 mV s^−1^ are presented in Figure 4a. All the NOC electrodes possess slight distorted rectangular-shaped CV plots with visible broad redox humps. This suggests that the capacitance of NOC is mainly contributed by outstanding EDLC that is related to its porous structure and that pseudo-capacitance came from the heteroatoms content. Ignoring the influence of the few heteroatoms in PAC-700 (The characterization of PAC-700 are shown in Figure S4) on its capacitance performance, the capacitance of PAC-700 could be roughly estimated as the EDLC effect. Comparing the CV curves of NOC-700 and PAC-700, the EDLC portion was calculated to be about 63.6%, by integrating the area of CV circles, and the pseudo-capacitance portion was determined to be 36.4%. This means that the pseudo-capacitance generated by a few N/O doping contributes significantly to the overall capacitance performance. Additionally, all the NOC electrodes shows much higher current densities than those of PAC-700, indicating that the additional N and O doping played an important role in the improvement of the capacitance behavior. In fact, it is difficult to distinguish the exact contributions of EDLC and pseudo-capacitance to an electrode, and this needs further research in the future.

The CV profile of NOC-700 samples presented the largest encircled area, followed by those of NOC-600 and NOC-800, which is due to its higher specific surface area and N/O doping content. This indicates that the capacitance performance was significantly influenced by the preparation temperature, which was closely related to the porous structure and doping of N/O, as discussed above. Figure 4b gives the CV curves of the NOC-700 electrode at sweep rates of 5 to 200 mV s^−1^. CV curves of NOC-600 and NOC-800 at different sweep rates are shown in Figure S5a,b. Even at a high sweep rate (200 mV s^−1^), nearly rectangular-shaped CV curves were found, indicating a rapid ion transfer capability and good rate of performance of NOC electrode materials.

Figure 4c provides a comparative representation of GCD plots of the prepared electrodes at a current density of 2 A g^−1^. The GCD curves of NOC-700, NOC-600, and NOC-800 electrodes, recorded at different current densities, are shown in Figure 4d and Figure S6a,b, respectively. The GCD curves exhibit linear and symmetrical shapes with a slight deviation from the isosceles triangle at the end of the discharge. This suggests a major EDLC behavior, with some Faradaic effects, of these electrodes, which is consistent with the CV tests above. Compared with the PAC electrode, significantly longer periods of total charge and discharge cycles were achieved by NOC electrode. Among them, NOC-700 could obtain the superior specific capacitance value of 414.2 F g^−1^ at 0.5 A g^−1^, which is better than many other heteroatoms-doping carbon nanostructured electrodes reported recently, such as N/O co-doped porous carbon from waterborne acrylonitrile copolymer (317.5 F g^−1^ at 0.5 A g^−1^) [47], O/N co-doped porous carbon from ammonium citrate (349 F g^−1^ at 1 A g^−1^) [31], N/P co-doped mesoporous graphene (349 F g^−1^ at 1 A g^−1^) [48], N/S co-doped hierarchical porous carbon from chitosan (322 F g^−1^ at 1 A g^−1^) [49], O/N co-doped porous carbon nanosheets (270 F g^−1^ at 0.5 A g^−1^) [41], etc. (more detailed information shown in Table S5). The significantly enhanced capacitance performance of NOC-700 can mainly be attributed to the following reasons. First, the high surface area of the materials could offer a certain amount of active sites for ion accumulation. Second, the hierarchical porous structure prefers rapid ions transportation that enhances the accessibility of active sites. Third, the co-doping of N/O is beneficial to the aqueous wettability and electrical conductivity of electrodes. Finally, the co-doping of N/O can provide additional pseudo-capacitance via Faradaic reactions, as analyzed above. It is worth noting that the co-doping amount of N and O in NOC-700 is not the highest according to the XPS results (Table S3), which means that the excellent porous characteristics of the electrode materials contribute more to the total capacitance. In particular, better pore size distribution and a higher surface area also favor the exposure of pseudo-capacitance active sites, resulting in a synergistic effect between EDLC and pseudo-capacitance.

Figure 4e gives the calculated specific capacitance and rate performance of different NOCs electrodes. The specific capacitance values decrease slightly with the increasing current density, attributed to the charge transfer resistance and electrical conductivity of the electrode materials themselves. Even at a high current density of 50 A g^−1^, the specific capacitance could be maintained at 267.5, 307.5, and 225.2 F g^−1^ for NOC-600, NOC-700, and NOC-800, corresponding to capacitance retentions of 72.0%, 74.2%, and 56.5%, respectively. These indicated that NOC electrodes possess excellent energy delivery capability in the case of quick charging and discharging. Another interesting bit of information is that the specific capacitance of NOC-800 is higher than that of NOC-600 at lower current densities, while, when the current density was raised to 5 A g^−1^, the specific capacitance of NOC-800 was gradually lower than that of NOC-600. This was due to the fact that NOC-800 possesses a larger surface area with a higher proportion of micro-pores than NOC-600. There is sufficient time for electrolyte ions to be transferred into the internal micro-pore structure at lower current densities, which can generate higher specific capacitances. However, the disadvantage of the poor porous distribution of NOC-800 was revealed at higher charging and discharging rate conditions. Its specific capacitance decreased rapidly due to the lacking of an efficient buffer zone and the quick transfer channel for electrolyte ions brought by macro- and meso-pores.

To evaluate the durability performance of the NOC-700 electrode, CV cycles tests were continuously carried out at 50 mV s^−1^ and are shown in Figure 4f. It is obvious that there is nearly no capacitance loss after 10,000 cycles, demonstrating that NOC-700 is a promising carbon electrode candidate for supercapacitors. Correspondingly, the CV curves of the NOC-700 electrode in the inset of Figure 4f shows almost no shrinking at the 5000th and 10,000th cycles. This indicates that the NOC-700 electrode material could maintain excellent structural stability over a long cycling period.

**Figure 5:**
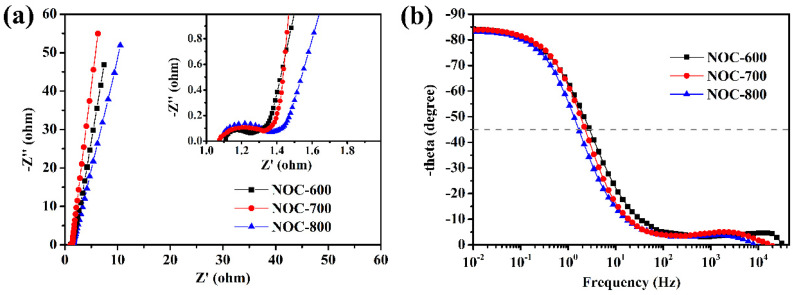
Nyquist plots (**a**) and Bode plots (**b**) of NOC materials. The inset is an enlarged image of the high frequency zone of Nyquist plots.

To further understand the electrochemical process of NOC electrodes, EIS analyses were carried out. The equivalent series resistance (ESR) of NOC electrodes, estimated by the intercept on the real axis of Nyquist plots in a high frequency zone were 1.11 Ω, 1.06 Ω, and 1.09 Ω for NOC-600, 700, and 800, respectively (Figure 5a), representing the rapid ion transport and diffusion due to the hierarchical porous structure of NOC materials. The small semicircle diameters detected at a medium frequency demonstrate the low charge transfer resistance of these three carbon materials, for both EDLCs and pseudo-capacitance effects. In addition, the Warburg impedance section in the transition zone from medium to high frequency is not obvious, which means that the electrolyte ion transport in NOC electrodes is so fast that the finite length capacitive effect could be almost ignored [31]. Importantly, the nearly vertical tails at low frequencies, indicating ideal capacitance performance, can be found in all NOC electrodes [50]. Among them, the NOC-700 electrode has the steepest line, indicating the best capacitance behavior, which coincides with the CV and GCD results.

As seen in the Bode phase plots (Figure 5b), the phase angle at a frequency of 0.01 Hz was −84.2°, −84.1°, and −83.1° for NOC-600, NPC-700, and NPC-800, respectively. These are close to the phase angle of −90° of an ideal capacitor. Relaxation time *τ*, which can be used to evaluate the discharge rate of electrodes, was determined by $\tau =1/f_{0}$ at a phase angle of −45° [51]. The constant τ value of NOC-600, NOC-700, and NOC-800, were 0.34 s, 0.43 s, and 0.60 s, respectively, implying that NOC-600 and NOC-700 are able to achieve a higher rate of capability than NOC-800 at larger charge–discharge current densities because of their better pore size distributions, which are beneficial to rapid ion transfers.

**Figure 6:**
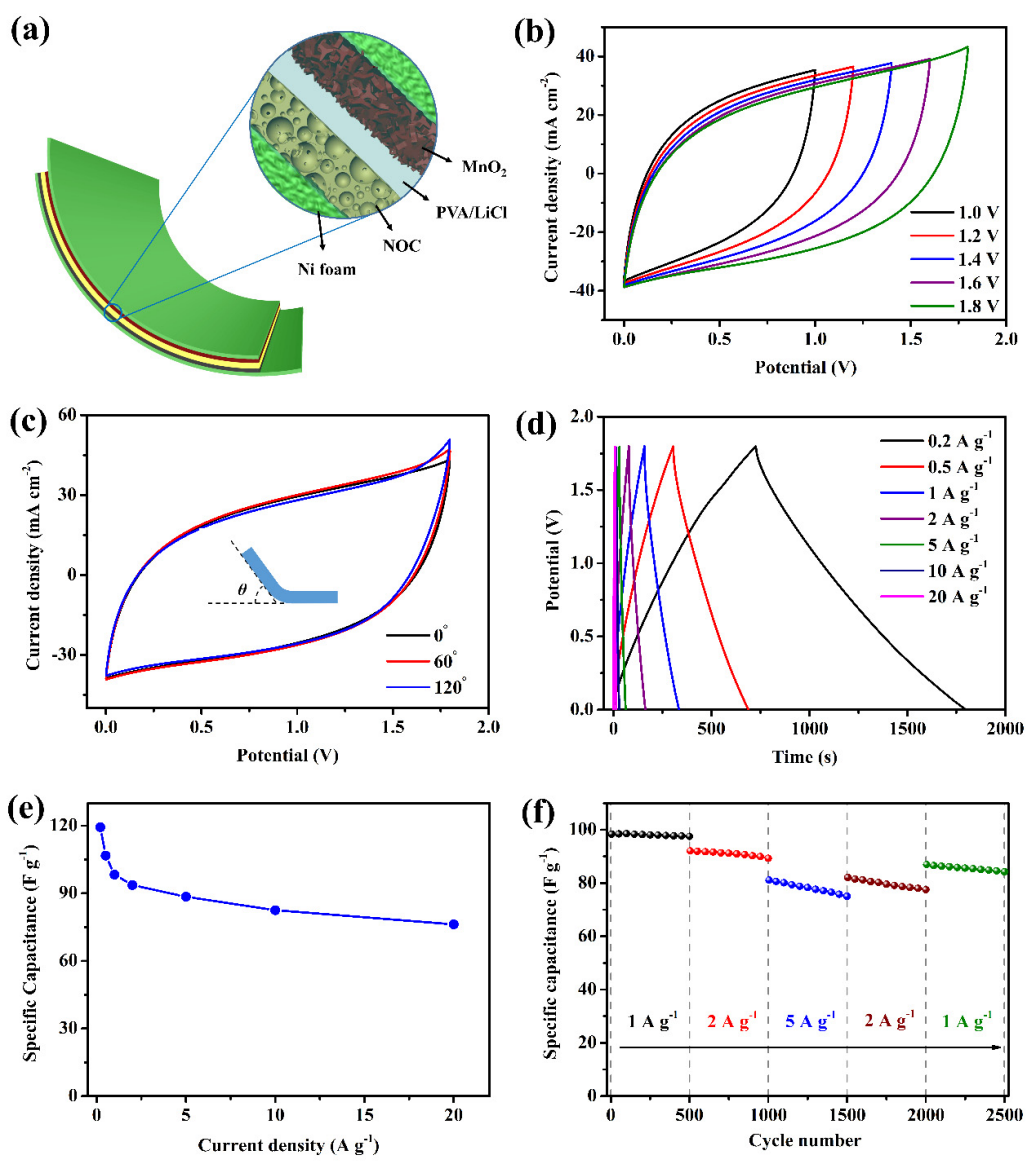
Scheme of the as-fabricated MnO_2_//NOC ASC device (**a**). CV curves of the ASC at different potential windows (**b**) and CV curves of different bending angles at 50 mV s^−1^ (**c**). The GCD curves at different current densities (**d**) at a voltage of 1.8 V and the corresponding specific capacitance (**e**). The cycling ability of ASC at different current densities (**f**).

The outstanding capacitance properties of NOC-700 further promotes the investigation of its practical application as an anode for supercapacitor devices. As shown in Figure 6a, an all-solid-state ASC device was fabricated using NOC-700 and *α*-MnO_2_ as the anode and cathode, respectively (denoted as MnO_2_//NOC ASC device). As is known, MnO_2_ is an excellent cathode material because of its outstanding capacitance performance, large effective potential range, and easy preparation [52,53]. The MnO_2_ electrode in this work was prepared by a direct electro-deposition method [54]. Details of the preparation method are presented in the Supplementary Materials. From the TEM images of the active materials of the MnO_2_ electrode (Figure S7a), it is clear that the MnO_2_ nanoparticles present an irregular accumulation. The typical Mn 2p XPS spectrum (Figure S7b) with a binding energy separation of 12.2 eV between Mn 2p_3/2_ and Mn 2p_1/2_ represents the existence of *α*-MnO_2_ [55].

The mass ratio of MnO_2_ and NOC active materials was calculated to be about 1.85:1 (detail method shown in the Supplementary Figure S8). Furthermore, CV tests were carried out on the optimized MnO_2_//NOC ASC device at different potential windows to determine the optimum operating conditions. As seen in Figure 6b, the stable working potential windows can be expanded to 0–1.8 V. Additionally, the all-solid-state ASC device possesses outstanding flexibility and mechanical stability under different bending angles of 0°, 60°, and 120° (Figure 6c) with almost same capacitance performance.

Moreover, the GCD curves of the MnO_2_//NOC ASC device (Figure 6d) exhibit a relative symmetric shape, indicating the superior charge–discharge behavior of ASC, which is also in keeping with the CV results. The corresponding specific capacitance of the device were shown in Figure 6e. A maximum capacitance of 119.3 F g^−1^ can be achieved at 0.2 A g^−1^. Additionally, an expected capacitance rate performance with a 63.9% initial capacitance retention (76.3 F g^−1^) was achieved as the current density increased to 20 A g^−1^. To further investigate the long-term durability of the ASC device, GCD tests were performed under various conditions. As illustrated in Figure 6f, the current densities were changed to other levels after every 500 cycles. The initial specific capacitance was calculated as 98.3 F g^−1^, 92 F g^−1^, and 81 F g^−1^ at 1 A g^−1^, 2 A g^−1^, and 5 A g^−1^, respectively, and the corresponding capacitance retentions after every 500 cycles were 99.2%, 96.9%, and 92.6%, respectively, which shows excellent cycling stability. In addition, the capacitance loss increases with an increase in current density. This indicates that a faster charging and discharging rate has a stronger destructive effect on the structure of electrode materials, especially the MnO_2_ cathode. Moreover, when the current densities were set back to 2 A g^−1^ and 1 A g^−1^ for another 500 cycles, the capacitance loss increased to 5.4% and 3.1%, respectively. On the one hand, this is because the electrode surface and gel electrolyte were damaged by the previous charging and discharging process; on the other hand, the higher current densities further aggravated such damage.

**Figure 7:**
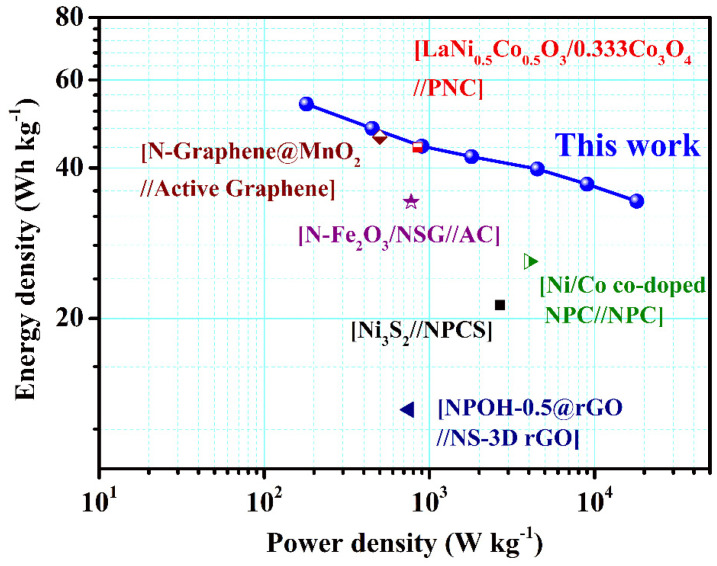
Ragone plot of as-fabricated MnO_2_//NOC ASC device.

The Ragone plot in Figure 7 demonstrates the relationship between energy density and power density of the as-fabricated MnO_2_//NOC ASC device. It shows an excellent energy density of 53.7 W h kg^−1^ at a power density of 180 W kg^−1^, which is higher than other carbon-based ASCs that have been recently reported, such as LaNi_0.5_Co_0.5_O_3_/0.333Co_3_O_4_//P/N co-doped carbon ASC (43.9 W h kg^−1^ at 850 W kg^−1^) [56], N-Fe_2_O_3_/NSG//AC (34.2 W h kg^−1^ at 775 W kg^−1^) [57], N-graphene@MnO_2_//active graphene (46.1 W h kg^−1^ at 500 W kg^−1^) [58], NPOH-0.5@rGO//NS-3D rGO (13.125 W h kg^−1^ at 750 W kg^−1^) [18], Ni/Co co-doped NPC//NPC (26 W h kg^−1^ at 4000 W kg^−1^) [43], Ni_3_S_2_//N doped CS (21.26 W h kg^−1^ at 2666.76 W kg^−1^) [59], etc. Even at a higher power density of 18.0 kW kg^−1^, this ASC device still showed an energy density of 34.3 W h kg^−1^, which represents its usefulness in practical energy storage devices.

## 4. Conclusions

In summary, we developed a sustainable and low-cost method to transform hazardous petroleum sludge waste into N/O co-doped porous carbon materials, which realize nanostructure engineering and heteroatom doping simultaneously with the assistance of KOH and urea. The as-synthesized NOC-700 sample had a hierarchical porous structure with a high surface area (2514.7 m^2^ g^−1^), balanced pore size distribution, and rich N/O-containing groups. Due to the synergistic effects of EDLC and pseudo-capacitance, which come from the rational porous structure and N/O doping, respectively, NOC-700 exhibited outstanding specific capacitance (414.2 F g^−1^ at 0.5 A g^−1^) and high-rate performance (74.2% retention at 50 A g^−1^). Moreover, the MnO_2_//NOC ASC device offered a high energy density of 53.7 W h kg^−1^ at 180 W kg^−1^ as well as excellent cycling stability, which suggests a strong possibility of its use in practical applications. Therefore, this work not only provides an efficient method for the development of a novel carbon material for energy storage, but also reveals a broad range of uses for the sustainable disposal and utilization of industrial solid wastes.

## Data Availability

Not applicable.

## Supplementary Materials

The following are available online at https://www.mdpi.com/article/10.3390/ma14102477/s1. Figure S1 SEM images of NOC-600 (a), NOC-800 (b), PSC (c) and PAC-700 (d); Figure S2 SEM images of NOC-650 (a) and NOC-750 (b); Figure S3 High resolution of C1s spectrum of NOC materials; Figure S4 The N2 adsorption–desorption isotherms (a), pore size distribution (b), XRD pattern (c) and XPS spectrum (d) of PAC material; Figure S5 CV curves of NOC-600 (a) and NOC-800 (b) at different sweep rates; Figure S6 GCD curves of NOC-600 (a) and NOC-800 (b) at different current densities; Figure S7 TEM image (a) and Mn 2p spectrum (b) of the MnO_2_ cathode material synthesized by electrodeposition approach. CV curves (c) of MnO_2_ cathode at different sweep rates in three-electrode system and the corresponding specific capacitance (d); Figure S8 The comparative CV curves of MnO_2_ and NOC-700 electrodes at 50 mV s^−1^; Table S1 Ultimate and proximate analyses of PS sample; Table S2 Porous parameters of N/O co-doped porous carbon materials; Table S3 XPS results of different NOCs samples; Table S4 Elemental content based on ultimate analysis; Table S5 Specific capacitance of PS-based NOC electrode versus recently published heteroatom-doped carbon electrodes.
