# Supplementary material for: Hazardous Petroleum Sludge-Derived Nitrogen and Oxygen Co-Doped Carbon Material with Hierarchical Porous Structure for High-Performance All-Solid-State Supercapacitors
Source: Materials (Basel). 2021 May 11;14(10):2477. doi: 10.3390/ma14102477 (PMC8151830; doi:10.3390/ma14102477)
Supplement: Supplementary file 1 [file materials-14-02477-s001.zip › materials-1169857-supplementary.pdf]

materials

MDPI

Supplementary Materials

Hazardous Petroleum Sludge-Derived Nitrogen and Oxygen Co-Doped Carbon Material with Hierarchical Porous Structure for High-Performance All-Solid-State Supercapacitors

Xiaoyu Li [1,3,*], Mingyang Zhang [2,*], Zhuowei Tan [3], Zhiqiang Gong [4], Peikun Liu [1] and Zhenbo Wang [3]

[1] College of Mechanical and Electronic Engineering, Shandong University of Science and Technology, Qingdao 266590, China; lpk@sdust.edu.cn
[2] School of Thermal Engineering, Shandong Jianzhu University, Jinan 250101, China
[3] College of New Energy, China University of Petroleum (East China), Qingdao 266580, China; b16030090@s.upc.edu.cn (Z.T.); wangzhb@upc.edu.cn (Z.W.)
[4] State Grid Shandong Electric Power Research Institute, Jinan 250003, China; gongzhiqiang@upc.edu.cn
* Correspondence: lixy2018@sdust.edu.cn (X.L.); zhangmingyang18@sdjzu.edu.cn (M.Z.)

Citation: Li, X.; Zhang, M.; Tan, Z.; Gong, Z.; Liu, P.; Wang, Z. Hazardous Petroleum Sludge-Derived Nitrogen and Oxygen Co-Doped Carbon Material with Hierarchical Porous Structure for High-Performance All-Solid-State Supercapacitors. *Materials* **2021**, *14*, 2477. https://doi.org/10.3390/ma14102477

Academic Editor: Barbara Gawdzik

Received: 19 March 2021
Accepted: 6 May 2021
Published: 11 May 2021

Publisher's Note: MDPI stays neutral with regard to jurisdictional claims in published maps and institutional affiliations.

Copyright: © 2021 by the authors. Licensee MDPI, Basel, Switzerland. This article is an open access article distributed under the terms and conditions of the Creative Commons Attribution (CC BY) license (http://creativecommons.org/licenses/by/4.0/).

Experimental Methods

Materials Characterization

Scanning electron microscopy (SEM, JEOL JSM-6700F, Japan) and transmission electron microscopy (TEM, JEOL-2010, Japan, 200 kV) were used to observe the surface morphology of materials. The X-ray diffraction (XRD, Panalytical X'pert Pro, Netherlands) and X-ray photoelectron spectroscopy (XPS, Thermo Fisher ESCALAB MK II, USA) test were performed to determine the composition and the surface elemental conditions of the samples. Then, N_2 adsorption-desorption experiment (Micromeritics APSP 2020, USA) were carried out to investigate the porous properties of the materials. The specific surface area and pore size distribution could be evaluated by Brunauer-Emmett-Teller (BET) method and Non-Local Density Functional Theory (NLDFT), respectively. The electrical conductivity was measured by a four-point probe resistivity meter (Beijing Heng Odd instrument).

Preparation of MnO_2 Cathode

The MnO_2 electrodes were prepared directly by the electro-deposition method [1]. In details, 0.01 M $Mn(CH_3COO)_2$ and 0.02 M $CH_3COO\ NH_4$ solution was prepared for electrolyte firstly. Then, a flexible Ni foam cleaned ultrasonically in distilled water, ethanol and acetone was used as working electrode. The Ni foam substrate was immersed into the electrolyte with a size of 1cm × 1cm. A graphite rod and a saturated calomel electrode with a double salt bridge were chosen as counter electrode and reference electrode, respectively. The above three-electrode system was fabricated for anodic electro-deposition of MnO_2 nanomaterials. The operation current density was set at 0.2 mA cm^{-2} for 150 min, and the solution temperature was controlled at 70 °C. The amount of MnO_2 deposition could be controlled by adjusting the electro-deposition time.

Preparation of PVA/LiCl Gel Electrolyte

To prepare PVA/LiCl gel electrolyte, 0.85 g LiCl (0.02 mol) and 2.0 g polyvinyl alcohol were mixed with 20.0 mL distilled water. Then, the mixture was heated to 90 °C and refluxed under vigorous magnetic stirring until the gel solution became clear.

Fabrication of MnO_2//NOC-700 ASC Devices

For a supercapasitor device, charge storage on the cathode and anode will be balance and follow the relationship of $Q_{cathode}=Q_{anode}$. The mass ratio of active material on both electrodes for the optimum performance satisfied the following equation,

$$\frac{m_{cathode}}{m_{anode}} = \frac{C_{anode} \times V_{anode}}{C_{cathode} \times V_{cathode}}$$

The overlaid CV curves of MnO_2 and NOC-700 measured on three electrode systems at a sweep rate of 50 mV s^{-1} are illustrated in Figure S6. As observed from the curves, the potential window of NOC-700 and MnO_2 is in the range of −1.0-0 V and 0–0.8 V, respectively.

Calculation Methods

Specific capacitance (C, F g^{-1}) of the single electrode or the ASC device could be calculated from the GCD curves based on the following equation:

$$C = \frac{I\Delta t}{m\Delta V}$$

Where I(A) is the discharge current, Δt (s) is the discharge time, ΔV (V) is the potential window, and m(g) is the mass of the active materials loaded on working electrode. For the ASC tests, m(g) represents the total mass of active materials on cathode and anode.

The energy density (E, W h kg^{-1}) and power density (P, W kg^{-1}) of the ASC device could be calculated by the following equations,

$$E = \frac{C\,V^2}{2 \times 3.6}$$

$$P = 3600 \times \frac{E}{\Delta t}$$

Where C and V represents the specific capacitance of the supercapacitor device and the testing potential windows, respectively.

Table S1. Ultimate and proximate analyses of PS sample.

**Items**	**Petroleum Sludge**				
**Ultimate analysis (*wt*%)**	**C**	**H**	**O**	N	S
	16.38	4.25	8.92	0.32	2.34
**Proximate analysis** (*wt*%)	Moisture[a]	Volatile matter[b]		Fixed carbon[a]	Ash[a]
	16.61	27.38		4.82	51.19

[a] As received.[b] Dry basis.

Table S2. Porous parameters of N/O co-doped porous carbon materials.

**Sample**	S_{BET} [m^2 g^{-1}]	S_{mirco} [m^2 g^{-1}]	S_{meso} [m^2 g^{-1}]	V_{pore} [cm^3 g^{-1}]	V_{mirco} [cm^3 g^{-1}]	V_{meso} [cm^3 g^{-1}]
NOC-600	767.7	290.8	476.9	0.956	0.127	0.829
NOC-700	2514.7	1059.4	1455.3	2.383	0.520	1.863
NOC-800	1847.0	1775.5	71.5	1.091	0.745	0.346

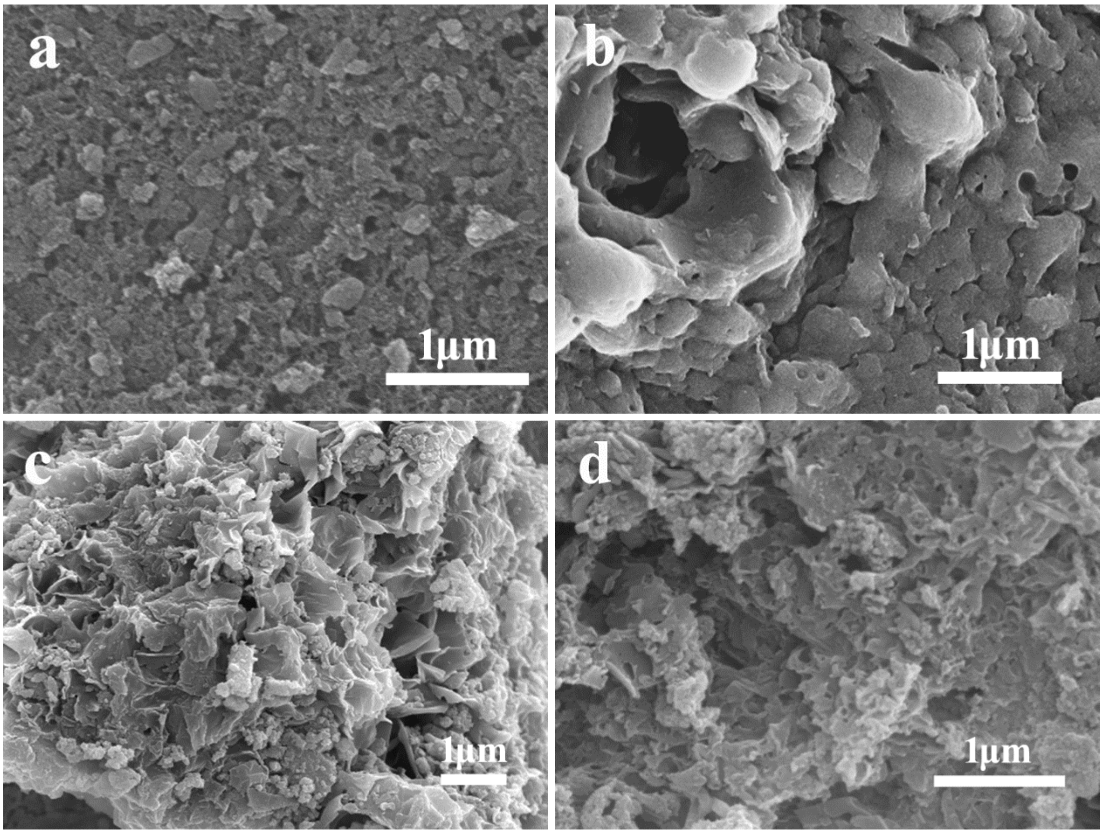

Figure S1. SEM images of NOC-600 (**a**), NOC-800 (**b**), PSC (**c**) and PAC-700 (**d**).

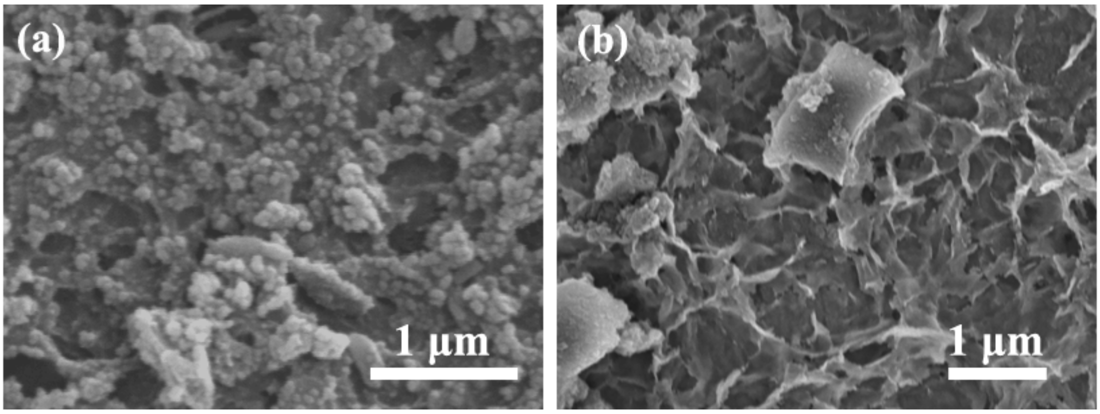

Figure S2. SEM images of NOC-650 (**a**) and NOC-750 (**b**).

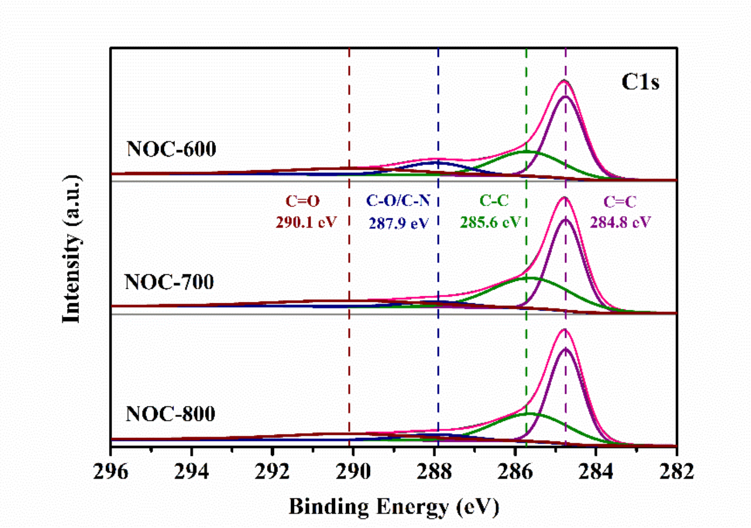

Figure S3. High resolution of C1s spectrum of NOC materials.

Table S3. XPS results of different NOCs samples.

Samples	C (at %)	O (at %)	N (at %)	Functionality (% of N1s)			
				N-5	N-6	N-Q	N-O
NOC-600	83.62	13.43	2.95	40.10	34.56	6.9	18.44
NOC-700	85.2	8.94	5.86	26.65	46.78	13.5	13.07
NOC-800	87.68	2.74	9.58	31.63	34.34	13.65	20.38

Table S4. Elemental content based on ultimate analysis.

Samples (wt %)	C	H	O	N
NOC-600	87.2	2.3	8.5	2.0
NOC-700	89.3	1.7	5.8	3.2
NOC-800	91.1	1.5	2.1	5.3

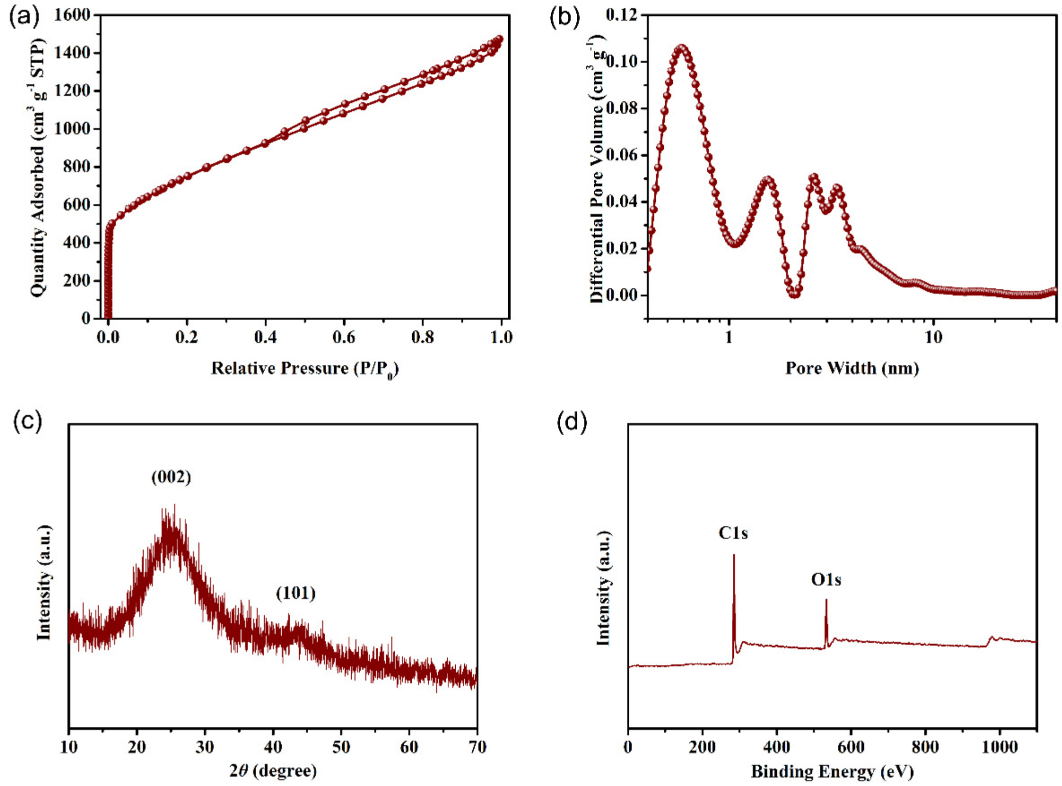

Figure 4. The N_2 adsorption-desorption isotherms (**a**), pore size distribution (**b**), XRD pattern (**c**) and XPS spectrum (**d**) of PAC material.

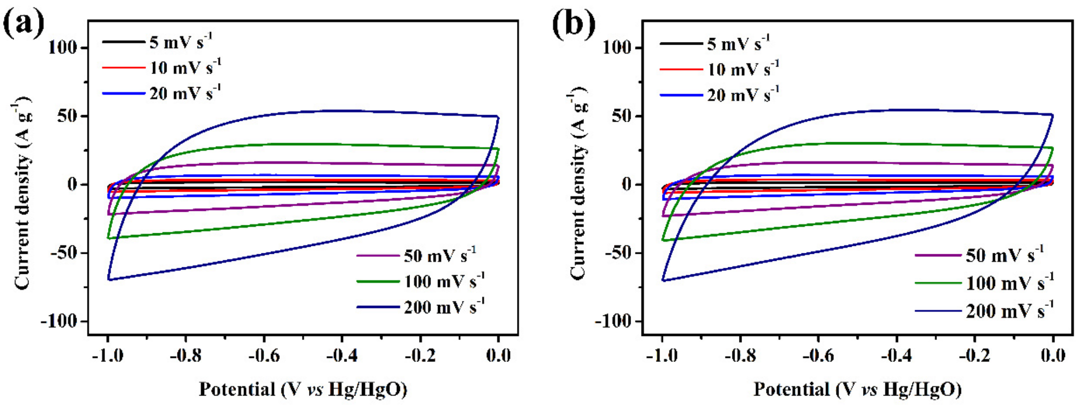

Figure S5. CV curves of NOC-600 (**a**) and NOC-800 (**b**) at different sweep rates.

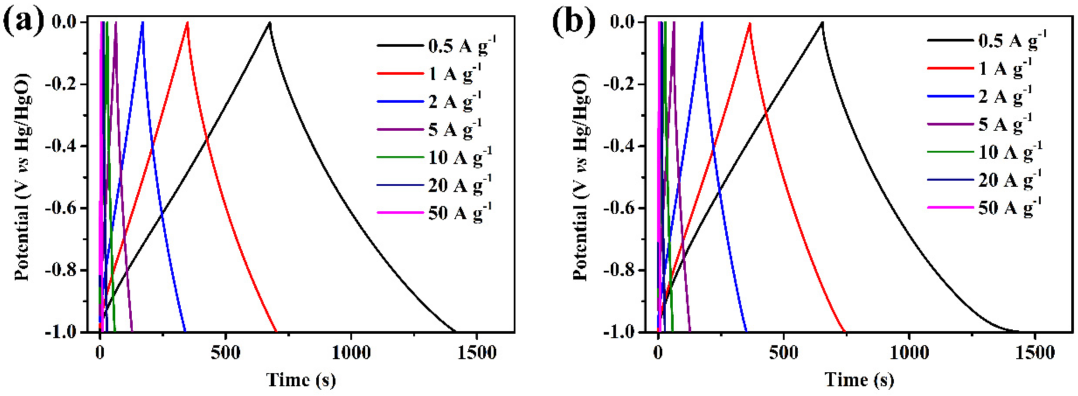

Figure S6. GCD curves of NOC-600 (**a**) and NOC-800 (**b**) at different current densities.

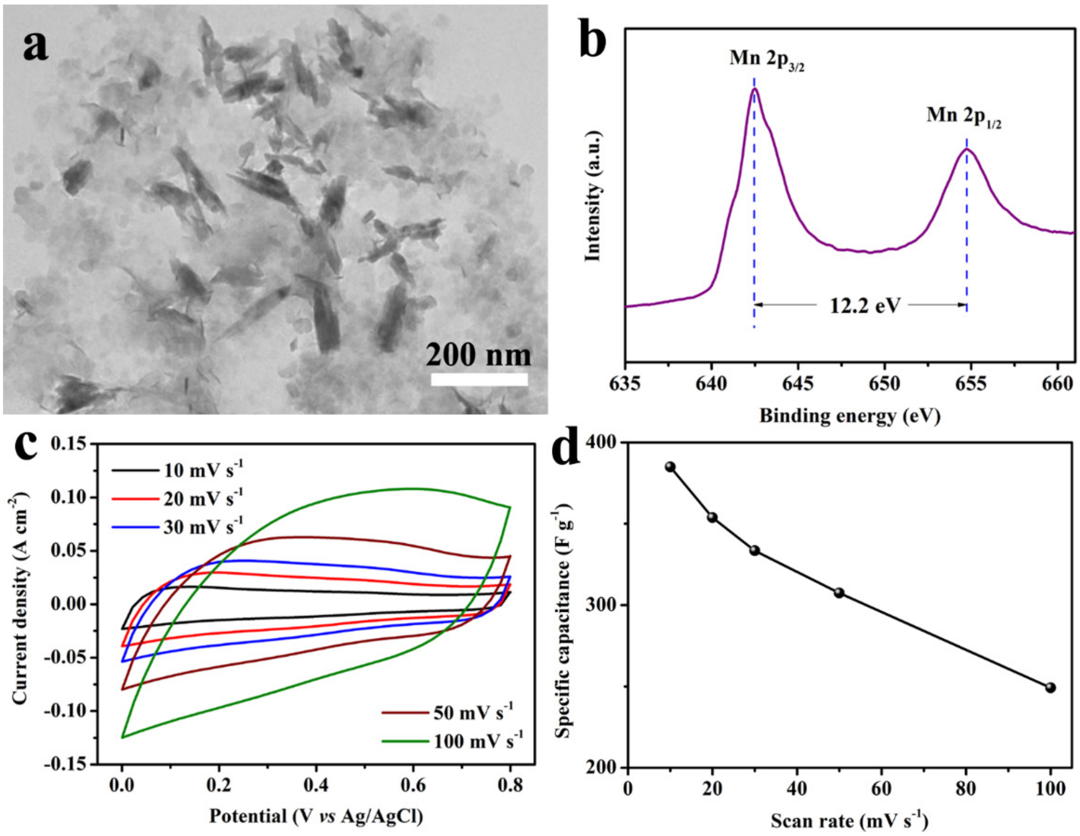

Figure S7. TEM image (**a**) and Mn 2p spectrum (**b**) of the MnO_2 cathode material synthesized by electrodeposition approach. CV curves (**c**) of MnO_2 cathode at different sweep rates in three-electrode system and the corresponding specific capacitance (**d**).

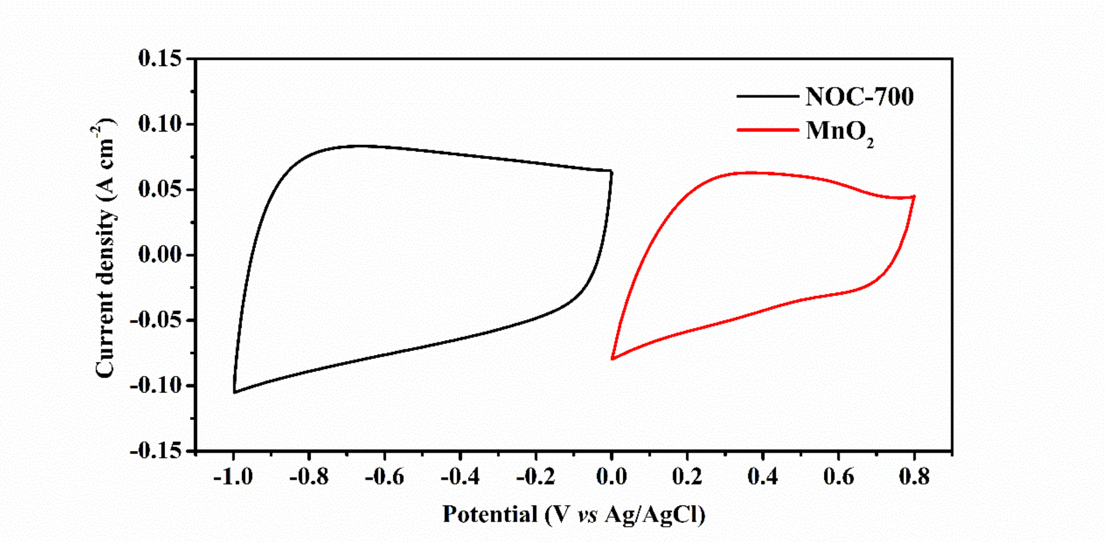

Figure S8. The comparative CV curves of MnO_2 and NOC-700 electrodes at 50 mV s^{-1}.

Table S5. Specific capacitance of PS-based NOC electrode versus recently published heteroatom-doped carbon electrodes.

Active Material	Electrolyte	Potential	Capacitance
NOC in this work	6 M KOH	−1.0~0 V *vs* Hg/HgO	414.2 F g^{-1} at 0.5 A g^{-1}
N/S co-doped graphene[2]	6 M KOH	−1.0~0 V *vs* Hg/HgO	208 F g^{-1} at 0.2 A g^{-1}

N/O co-doped carbon networks[3]	6 M KOH	−0.95~0 V *vs* SCE	435 F g^{-1} at 0.1 A g^{-1}
O/N co-doped porous carbon[4]	6 M KOH	−1.0~0 V *vs* Hg/HgO	270 F g^{-1} at 0.5 A g^{-1}
N/S/O co-doped hierarchical porous carbon[5]	6 M KOH	−1.0~0 V *vs* SCE	367 F g^{-1} at 0.3 A g^{-1}
N/S co-doped porous carbon nanosheets[6]	6 M KOH	−1.0~0 V *vs* Ag/AgCl	298 F g^{-1} at 0.5 A g^{-1}
B/N/O co-doped carbon[7]	6 M KOH	−1.0~0 V *vs* Hg/HgO	238 F g^{-1} at 0.5 A g^{-1}
N/O co-doped carbon sponge[8]	6 M KOH	−1.0~0 V *vs* Ag/AgCl	242 F g^{-1} at 0.5 A g^{-1}
N/P co-doped porous carbon[9]	6 M KOH	−1.0~0 V *vs* SCE	315.2 F g^{-1} at 1 A g^{-1}
N-doped porous carbon[10]	6 M KOH	−1.0~0 V *vs* Hg/HgO	365 F g^{-1} at 0.5 A g^{-1}
N-doped porous carbon nanofibers[11]	6 M KOH	−1.0~0 V *vs* Hg/HgO	202.0 F g^{-1} at 1 A g^{-1}
O/N co-doped porous carbon[12]	6 M KOH	−1.0~0 V *vs* SCE	187 F g^{-1} at 0.5 A g^{-1}
O/N co-doped carbon nanofibers[13]	6 M KOH	−1.0~0 V *vs* Ag/AgCl	233.1 F g^{-1} at 0.2 A g^{-1}
P/N co-doped porous carbon[14]	6 M KOH	−1.0~0 V *vs* Hg/HgO	318 F g^{-1} at 1 A g^{-1}
N/P co-doped mesoporous graphene[15]	6 M KOH	−1.0~0 V *vs* Hg/HgO	245 F g^{-1} at 0.5 A g^{-1}
N/O co-doped porous carbon[16]	6 M KOH	−1.0~0 V *vs* Hg/HgO	187 F g^{-1} at 0.5 A g^{-1}

Reference

1. Lu, X.; Zheng, D.; Zhai, T.; Liu, Z.; Huang, Y.; Xie, S.; Tong, Y. Facile synthesis of large-area manganese oxide nanorod arrays as a high-performance electrochemical supercapacitor. *Energy Environ. Sci.* **2011**, *4*, 2915–2921, doi:10.1039/c1ee01338f.
2. Cheng, L.; Hu, Y.; Qiao, D.; Zhu, Y.; Wang, H.; Jiao, Z. One-step radiolytic synthesis of heteroatom (N and S) co-doped graphene for supercapacitors. *Electrochim. Acta* **2018**, *259*, 587–597, doi:10.1016/j.electacta.2017.11.022.
3. He, D.; Niu, J.; Dou, M.; Ji, J.; Huang, Y.; Wang, F. Nitrogen and oxygen co-doped carbon networks with a mesopore-dominant hierarchical porosity for high energy and power density supercapacitors. *Electrochim. Acta* **2017**, *238*, 310–318, doi:10.1016/j.electacta.2017.03.218.
4. 4. Liu, B.; Liu, Y.; Chen, H.; Yang, M.; Li, H. Oxygen and nitrogen co-doped porous carbon nanosheets derived from Perilla frutescens for high volumetric performance supercapacitors, *J. Power Sources* **2017**, *341*, 309–317.
5. Yang, W.; Yang, W.; Song, A.; Gao, L.; Su, L.; Shao, G. Supercapacitance of nitrogen-sulfur-oxygen co-doped 3D hierarchical porous carbon in aqueous and organic electrolyte. *J. Power Sources* **2017**, *359*, 556–567, doi:10.1016/j.jpowsour.2017.05.108.
6. Li, Y.; Wang, G.; Wei, T.; Fan, Z.; Yan, P. Nitrogen and sulfur co-doped porous carbon nanosheets derived from willow catkin for supercapacitors. *Nano Energy* **2016**, *19*, 165–175, doi:10.1016/j.nanoen.2015.10.038.
7. Wang, C.; Zhang, X.; Wang, J.; Ma, Y.; Lv, S.; Xiang, J.; Chu, M.; Sun, T.; Qin, C. Boron/Nitrogen/Oxygen Co-Doped Carbon with High Volumetric Performance for Aqueous Symmetric Supercapacitors. *J. Electrochem. Soc.* **2018**, *165*, A856–A866, doi:10.1149/2.0321805jes.
8. Zhang, R.; Jing, X.; Chu, Y.; Wang, L.; Kang, W.; Wei, D.; Li, H.; Xiong, S. Nitrogen/oxygen co-doped monolithic carbon electrodes derived from melamine foam for high-performance supercapacitors. *J. Mater. Chem. A* **2018**, *6*, 17730–17739, doi:10.1039/c8ta06471g.
9. Wang, T.; Zhang, J.; Hou, Q.; Wang, S. Utilization of nutrient rich duckweed to create N, P Co-doped porous carbons for high performance supercapacitors. *J. Alloy. Compd.* **2019**, *771*, 1009–1017, doi:10.1016/j.jallcom.2018.08.295.
10. Tan, J.; Chen, H.; Gao, Y.; Li, H. Nitrogen-doped porous carbon derived from citric acid and urea with outstanding supercapacitance performance, *Electrochimica Acta* **2015**, *178*, 144–152.
11. Chen, L.-F.; Zhang, X.-D.; Liang, H.-W.; Kong, M.; Guan, Q.-F.; Chen, P.; Wu, Z.-Y.; Yu, S.-H. Synthesis of Nitrogen-Doped Porous Carbon Nanofibers as an Efficient Electrode Material for Supercapacitors. *Acs Nano* **2012**, *6*, 7092–7102, doi:10.1021/nn302147s.
12. Chen, W.; Luo, M.; Liu, C.; Hong, S.; Wang, X.; Yang, P.; Zhou, X. Fast microwave self-activation from chitosan hydrogel bead to hierarchical and O, N co-doped porous carbon at an air-free atmosphere for high-rate electrodes material. *Carbohydr. Polym.* **2019**, *219*, 229–239, doi:10.1016/j.carbpol.2019.05.033.
13. Li, Q.; Xie, W.; Liu, D.; Wang, Q.; He, D. Nitrogen and oxygen co-doped carbon nanofibers with rich sub-nanoscale pores as self-supported electrode material of high-performance supercapacitors. *Electrochim. Acta* **2016**, *222*, 1445–1454, doi:10.1016/j.electacta.2016.11.123.
14. Wang, N.; Wang, C.; He, L.; Wang, Y.; Hu, W.; Komarneni, S. Incomplete phase separation strategy to synthesize P/N co-doped porous carbon with interconnected structure for asymmetric supercapacitors with ultra-high power density. *Electrochim. Acta* **2019**, *298*, 717–725, doi:10.1016/j.electacta.2018.12.145.

15. Wang, X.; Liu, Y.; Wu, P. Water-soluble triphenylphosphine-derived microgel as the template towards in-situ nitrogen, phosphorus co-doped mesoporous graphene framework for supercapacitor and electrocatalytic oxygen reduction. *Chem. Eng. J.* **2017**, *328*, 417–427, doi:10.1016/j.cej.2017.07.064.
16. Lu, Y.; Chen, N.; Bai, Z.; Mi, H.; Ji, C.; Sun, L. Acid-Assisted Strategy Combined with KOH Activation to Efficiently Optimize Carbon Architectures from Green Copolymer Adhesive for Solid-State Supercapacitors. *Acs Sustain. Chem. Eng.* **2018**, *6*, 14838–14846, doi:10.1021/acssuschemeng.8b03377.
